# Minimal short-term decline in functional performance and quality of life predicts better long-term outcomes for both in older Taiwanese adults after hip fracture surgery: a prospective study

**DOI:** 10.1186/s13018-023-04278-3

**Published:** 2023-10-24

**Authors:** Tzu-I Yang, Yi-Jie Kuo, Shu-Wei Huang, Yu-Pin Chen

**Affiliations:** 1 Department of Orthopedic Surgery, Changhua Christian Hospital, Changhua, Taiwan; 2 Department of Orthopedics, Wan Fang Hospital, Taipei Medical University, No. 111, Sec. 3, Xinglong Rd., Wenshan Dist., Taipei City, 116 Taiwan; 3 Department of Orthopedics, School of Medicine, College of Medicine, Taipei Medical University, Taipei, Taiwan; 4 Graduate Institute of Health and Biotechnology Law, Taipei Medical University, Taipei, 116 Taiwan

**Keywords:** Activities of daily living, Quality of life, Hip fracture, Older adult

## Abstract

**Background:**

Hip fracture can lead to long-term loss of mobility and self-care ability in older adults. Despite initial decreases in functional performance after hip fracture surgery, patients tend to gradually recover. However, recovery can vary, with some regaining their abilities quickly while others becoming functionally dependent. In this study, we investigated whether the level of short-term postoperative decline in activity of daily living (ADL) performance and quality of life (QoL) can predict the 1-year outcomes for both following hip fracture surgery in older Taiwanese adults.

**Methods:**

This prospective cohort study included 427 older adults (≥ 60 years) who underwent hip fracture surgery at a single tertiary medical center in Taiwan between November 2017 and March 2021. We collected pre-fracture data, including the patients’ demographics, Charlson comorbidity index (CCI) scores, and responses to a questionnaire (Short Portable Mental State Questionnaire [SPMSQ]) for dementia screening. Moreover, their scores on the EuroQol-5D questionnaire (for evaluating QoL) and the Barthel Index (for assessing ADL performance) were collected at pre-fracture status and at 3- and 12-months following surgery. Changes in ADL and QoL three months post-surgery compared to pre-fracture status were evaluated, and the associations of these parameters (and other potential factors) with 1-year outcomes for ADL and QoL were investigated.

**Results:**

We analyzed the data of 318 patients with hip fracture and complete follow-up data regarding ADL performance and QoL at 3- and 12-months post-surgery. After adjusting for covariates, multivariate linear regression revealed that changes in ADL and QoL at 3 months post-surgery from pre-fracture status were positively and significantly associated with 1-year outcomes for both (*p* < .001 for both). Furthermore, pre-fracture CCI and SPMSQ scores were independent predictive factors associated with 1-year ADL outcomes (*p* = .042 and < .001, respectively).

**Conclusions:**

Patients who exhibit a smaller decline in functional performance and quality of life three months after hip fracture surgery from pre-fracture status are likely to have improved long-term ADL and QoL.

*Trial registration***:** TMU-JIRB N201709053.

**Supplementary Information:**

The online version contains supplementary material available at 10.1186/s13018-023-04278-3.

## Introduction

Hip fracture is a major concern for the older adults. It is associated with high postoperative mortality and morbidity rates, as well as substantial healthcare costs [1, 2]. Despite the decreasing incidence global rate of hip fracture, the number of cases has increased in some Asian countries [3]. The number of hip fracture cases in Asia is estimated to increase from 1,124,060 in 2018 to 2,563,488 by 2050, with a corresponding increase (from 9.5 to 15 billion US dollars) in the direct cost of hip fracture treatment [4].The risk of hip fracture has been increasing with the age of the population[5]; thus, clinicians must adopt various measures and effective treatment protocols to prevent a poor prognosis in older adults with hip fracture.

Our previous research found that one-third of older patients with hip fracture experience severe dependence on daily activities a year after surgery[6]. We also identified pre-fracture performance in daily activities as a critical predictor of both quality of life (QoL) and long-term functional outcomes [6]. In another study, we prospectively investigated the changes in the activity of daily living (ADL) and quality of life (QoL) within 1 year after hip fracture surgery in older patients [7]. The patients exhibited the poorest ADL performance and QoL 3 months postoperatively; nevertheless, gradual improvements were noted at 1-year follow-up, but their functional performance and QoL remained poorer than they were before the hip fracture [7]. Hip fracture may result in long-lasting effect on functional loss and reduced quality of life in the older adults. This raises our concern regarding the determining factors that influence a patient’s recovery.

The variability in recovery among older adults from physical stressors like illness or injury is well known. Physical resilience, which refers to the ability to endure or recover from functional decline caused by acute or chronic health stressors [8], may play a key role in older adults' recovery from significant stressors. Greater resilience could result in better recovery from major illnesses and enhance predictions of functional recovery[9]. One study divided patients into three resiliency groups after a hip fracture and found that pre-fracture function was the strongest predictor of high resilience [10]. Furthermore, another study recognized both ADL and QoL as appropriate tools for evaluating physical resilience [11]. This aligns with our prior research that found pre-fracture performance in daily activities is a critical factor in long-term functional outcomes [6]. On the other hand, considering the quick and complete recoveries commonly observed in older adults with high physical resilience, we hypothesized that patients who have a smaller degree of functional loss three months after hip fracture surgery may have better long-term outcomes. This hypothesis can serve as a useful clinical reference in advocating for early postoperative interventions, such as multidisciplinary rehabilitation, to enhance long-term outcomes for older adults who underwent hip fracture surgery [12]. However, evidence on the links between short- and long-term outcomes is scarce.

On the basis of potential prognostic factors in the older adults, a stratified care approach can be developed for providing intensive care to older patients with hip fracture who have a high risk of poor outcomes [13]. Several factors, such as age, sex, cognitive impairment, and pre-fracture Charlson comorbidity index (CCI) score, serum hemoglobin level, serum albumin level, and surgical factors including delay in operation for predicting the functional outcomes of hip fracture surgery in older patients exhibit high variance [14–17]. The present study aimed to evaluate the impact of short-term functional changes from pre-fracture on long-term outcomes and compare their association with other established clinical predictors. We examined changes in ADL performance and QoL at 3 months after hip fracture surgery in older Taiwanese patients and compared the predictive value of these factors with other independent factors for 1-year outcomes.

## Patients and methods

### Study design

This prospective cohort study included 427 older adults who underwent hip fracture surgery at a single medical center, Wan Fang Hospital, Taipei, Taiwan, between November 2017 and March 2021. The inclusion criteria were age > 60 years and a diagnosis of hip fracture, including intracapsular proximal femoral neck fracture and extracapsular (i.e., basal neck, subtrochanteric, or intertrochanteric) fracture. The patients underwent hip hemiarthroplasty or open reduction with internal fixation (with intramedullary nails or in situ cannulated or dynamic hip screws). Patients were excluded if they had undergone hip fracture surgery because of a primary condition other than hip fracture, such as avascular necrosis of the femoral head, infection, tumor metastasis, trauma, or osteoarthritis.

All patients received standard postoperative care and in-home rehabilitation after surgery, but the actual approach was tailored to each individual, taking into account their specific conditions and the preferences of the experts overseeing their care. Recovery efforts began promptly after hip fracture surgery, usually within 24 h, with the main objectives of preserving pre-fracture strength and preventing complications associated with immobility [18]. Early mobilization is prioritized, such as sitting in a chair shortly after surgery to minimize pressure sores, blood clots, and to aid in standing up. Patients receive instructions for daily exercises targeting the core, arms, and legs to strengthen their muscles. Over time, they are gradually encouraged to shift weight onto the uninjured leg, starting from the first day. Ambulation exercises begin after 4 to 8 days, assuming individuals can comfortably bear their full weight and maintain balance. The rehabilitation program may also incorporate stair-climbing exercises, fall prevention techniques, and guidance on using assistive devices like canes. During hospitalization, all patients receive training from professional physical therapists, and within the first three months after discharge.

Following discharge, the physical therapy facet of the intervention is designed to pinpoint and rectify issues tied to the strength of the upper and lower extremities, balance, transitioning between positions, walking, and stair climbing. The interventions aim to enhance walking proficiency, the ability to shift between positions, and bed mobility. This encompasses educating participants on safer and more effective techniques, providing assistance with using assistive tools, and implementing environmental adaptations. These in-home rehabilitation programs are all overseen by skilled physical therapists and adhere to the principles of systematic home-based physical and functional therapy as outlined in the publication by Tinetti et al. [19]. Regular monthly check-ups are conducted to evaluate the progress and effectiveness of home rehabilitation, as well as to offer pertinent guidance. This study was approved by the Ethics Committee of Taipei Medical University (TMU-JIRB N201709053). All participants provided written informed consent for participation in this study and the publication of their data.

### Evaluation of clinical parameters

We collected data regarding the patients’ basic characteristics, including age, sex, body mass index (BMI) and pre-fracture residence (home or care institution from their medical records as well as preoperative laboratory data, such as the levels of hemoglobin, serum sodium, creatinine, and albumin—predictors of hip fracture surgery outcomes in older patients [7, 20, 21]. CCI scores were determined to reflect the patients’ underlying comorbidities. In addition, we assessed surgical delay, which is the period between a patient’s falling accident and surgery. From the surgical records, we extracted the following information: hip fracture type (femoral neck or periprosthetic), previous contralateral hip fracture, surgical methods (internal fixation and hemiarthroplasty), intraoperative blood loss, and surgery duration. Furthermore, we interviewed the patients or their caregivers to obtain the patients’ pre-fracture data (upon admission for surgery). Their handgrip strength was assessed to screen for sarcopenia, and the Short Portable Mental Status Questionnaire (SPMSQ) was administered to screen for dementia [22]; the aforementioned conditions may influence patients’ physical function recovery [23]. All assessments were performed by a clinician within 1 week of admission.

The primary outcomes measured one year after hip fracture surgery were activities of daily living (ADL) and quality of life (QoL), evaluated using the Barthel Index (BI) and EuroQol-5D-3L (EQ-5D-3L) questionnaire, respectively [24, 25]. The pre-fracture status of EQ-5D-3L scores and BI was recorded at admission through a report by the patient or their caregiver. EQ-5D-3L scores and BI were reassessed through telephone interviews at 3- and 12-month postoperative follow-ups. We defined changes in Activities of Daily Living (ADL) and Quality of Life (QoL) at 3 months post-surgery compared to the pre-fracture status by subtracting the EQ-5D-3L and BI scores at the pre-fracture status from the scores at 3 months after surgery, respectively. And this was the main focus of the study to investigate the association with one-year outcomes.

### Instruments

The EQ-5D-3L questionnaire is an international instrument used for assessing health-related QoL [25]. We used the three-level version of the EQ-5D-3L questionnaire (EQ-5D-3L), which evaluates patients’ health-related QoL as having no problems, some problems, or major problems. The five dimensions of this questionnaire are mobility, self-care, usual activities, pain/discomfort, and anxiety/depression. The scale is numbered from 0 to 100, with “100” means the best heath and “0” means the worst health. The value attributed to EQ-5D-3L profile was presented based on a set of weights that reflect. The value is anchored at 1(representing full health) and 0 (indicating a state as serve as being dead) [25]. In the present study, the Chinese version of the EQ-5D-3L questionnaire was used, which has exhibited high levels of agreement (intraclass correlation coefficient > 0.75) and relatedness (convergent validity; Pearson’s correlation coefficient > 0.95) with the versions from the United Kingdom, Japan, and Korea [26].

The BI is used to evaluate individuals’ mobility and performance in ADLs. Total scores on this 10-item ordinal scale range from 0 to 100 [24]. A higher score (BI) indicates greater functional independence. The BI is also used to assess functional recovery in patients who undergo hemiarthroplasty after a femoral neck fracture [27]. The Chinese version of this scale exhibited moderate to excellent validity (intraclass correlation coefficient = 0.94) and interrater reliability (kappa value = 0.53–0.94) [28].

The SPMSQ is a 10-item cognitive screening instrument. The questionnaire is used to obtain data regarding individuals’ temporal and spatial orientation, memory, basic knowledge (date, day of the week, name of their current location, phone number, date of birth, age, names of the present and previous ministers, and name of their mothers), and ability to perform simple calculations (counting backward from 20 by 3’s). The total score ranges from 0 to 10, and a score of ≥ 3 indicates a clinical or neuropsychological condition [29]. The total number of errors is correlated with a clinical diagnosis of organic brain impairment or dementia. The Chinese version of this scale was validated in an earlier study (Cronbach’s alpha = 0.69) [22].

### Measurement of handgrip strength

The patients’ maximum isometric handgrip strength was measured using a handheld dynamometer (Sammons Preston, Bolingbrook, IL, USA). The participants were instructed to squeeze the device three times as hard as they possibly could using one hand while sitting with their elbow flexed and wrist in a neutral position [30]. The assessor was blinded to the patients’ reports. The Asian Working Group for Sarcopenia defines low handgrip strength as < 28 kg for men and < 18 kg for women. [31].

### Statistical analysis

Statistical analyses were performed using SPSS Statistics (version 22; IBM Corporation, Armonk, NY, USA). Categorical variables are presented as frequencies and percentage values, whereas continuous variables are presented in terms of means ± standard deviations. Univariate analyses were performed for identifying potentially significant predictors of 1-year surgical outcomes. Due to distribution of the EQ-5D-3L scores and BI, the normality, using the K-S test, assumption may be violated; as a result, the log transformation was conducted for ADL performance and QoL to strengthen normality.

The Mann–Whitney U test was used to compare continuous variables. Pearson correlation analysis was performed to analyze the correlation between the patients’ ADL performance and QoL 1 year after surgery and the potential predictors identified in the univariate analyses. Additionally, the correlation between the BI and EQ5D scores before surgery, at 3 months postoperatively, and at 1 year postoperatively was investigated. The predictors that differed significantly (*p* < 0.05) in the univariate analyses were included in multivariate linear regression models to calculate the beta coefficient with 95% confidence intervals. Two linear regression models were constructed, one each with the patients’ ADL performance and QoL at 1-year follow-up as the dependent variable. In addition, patients were classified into three groups (improvement/maintenance, decline < 50%, decline > 50%) based on changes in ADL and QOL three months post-surgery compared to their pre-fracture status. To detect differences in 1-year outcomes following hip fracture surgery, an Analysis of Variance (ANOVA) was conducted followed by a post hoc test with Bonferroni correction. Statistical significance was set at *p* < 0.05.

## Results

**Fig. 1:**
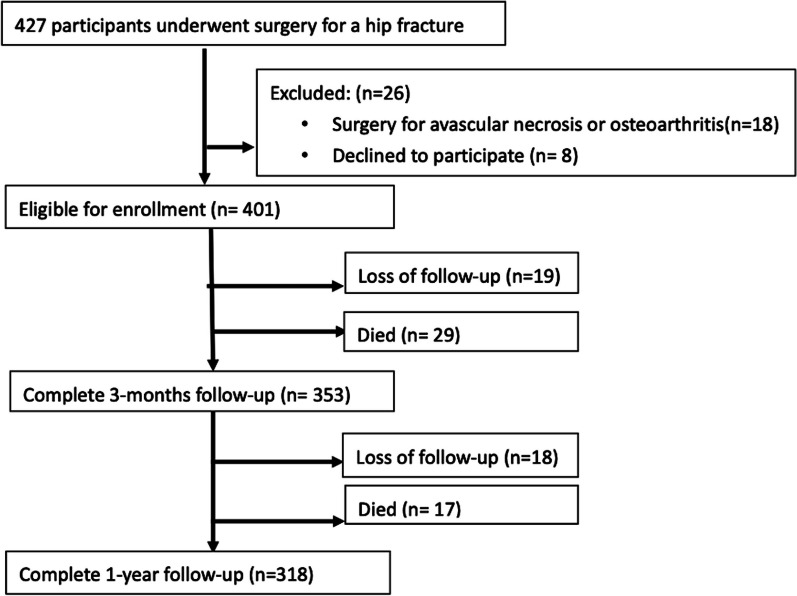
Flowchart for participant enrollment

**Fig. 2:**
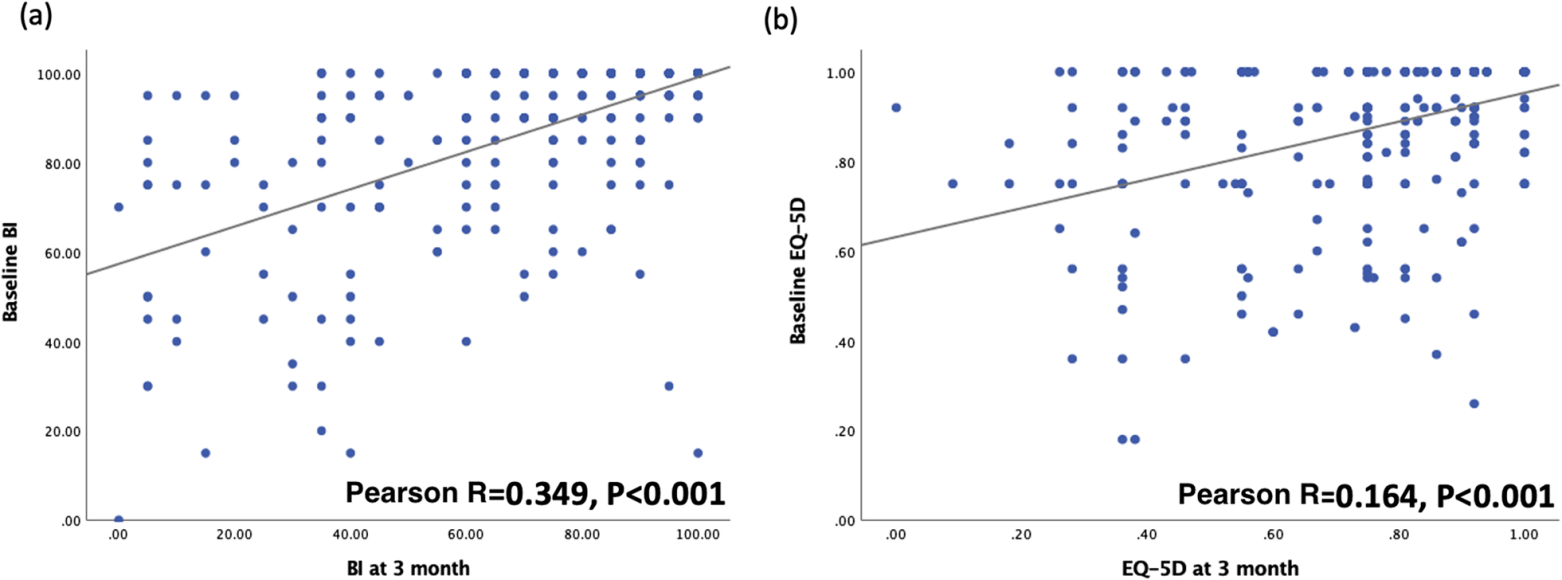
Pearson correlation coefficients for baseline score and 3-month outcomes. **a** Barthel Index and **b** EuroQol-5D-3L

**Fig. 3:**
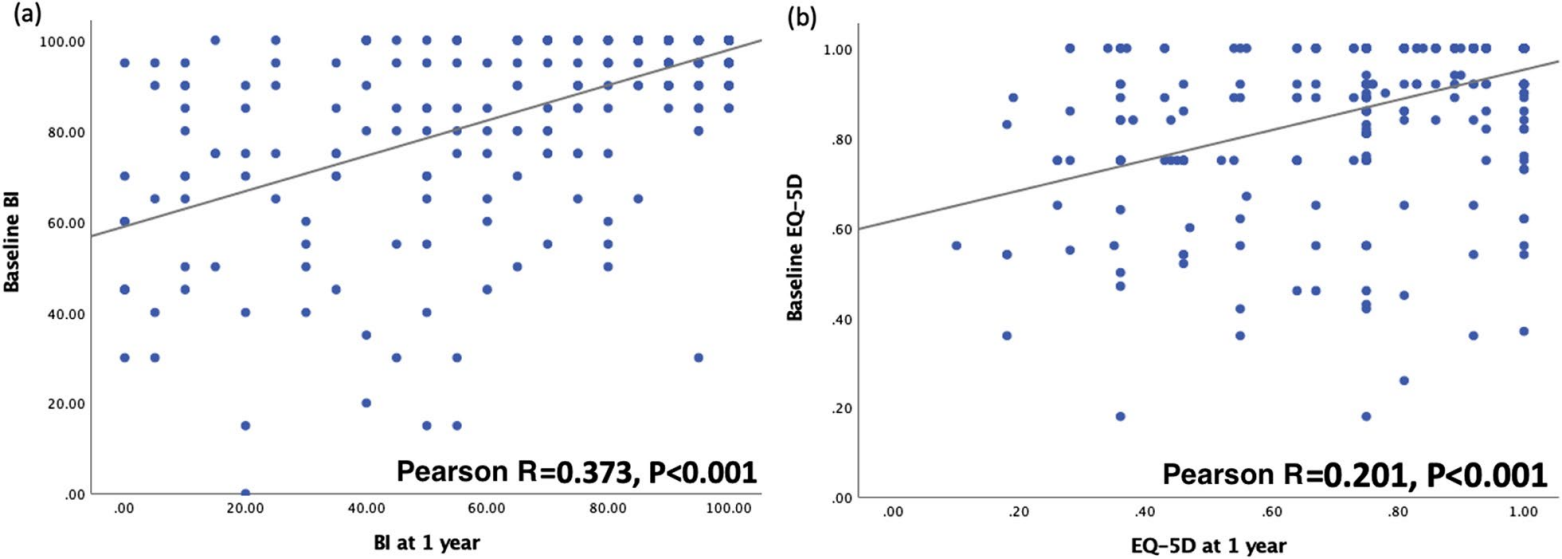
Pearson correlation coefficients for baseline score and 1-year outcomes. **a** Barthel Index and **b** EuroQol-5D-3L

Overall, 401 patients with hip fracture were eligible for enrollment (Fig. 1). In total, 83 participants died or were lost to follow-up within 1 year of hip fracture surgery. Thus, we analyzed the data of 318 patients (men, 97 [30.5%]; mean age, 80.23 ± 9.29 years). Table 1 summarizes the patients’ basic characteristics. Among the patients, 58.5% had femoral neck fractures and 41.5% had pertrochanteric fracture. 4.1% of the patients experienced a contralateral hip fracture. Furthermore, 182 patients (57.2%) received open reduction and internal fixation, while the remaining (42.8%) patients underwent hemiarthroplasty. The majority of patients, 92.1%, live in a home before hip fracture. The patients’ mean BMI was 22.37 ± 3.85 kg/m^2^. Upon admission, the mean SPMSQ score was 2.98 ± 3.56, handgrip strength was 12.88 ± 8.52 kg, CCI score was 4.75 ± 1.61, and surgical delay was 3.64 ± 9.03 day. Their mean BI score was 87.99 ± 19.30 at pre-fracture, 73.38 ± 27.28 at the 3-month follow-up, and 74.62 ± 30.24 at the 1-year follow-up. The EQ-5D-3L score was 0.88 ± 0.17 at pre-fracture, 0.78 ± 0.22 at the 3-month follow-up, and 0.79 ± 0.23 at the 1-year follow-up. Figures 2 and 3 illustrate the correlations between the preoperative baseline BI and EQ-5D score and at 3 months, as well as 1 year after the operation, respectively. Both of them were positively associated with the outcomes at 3 months and 1 year after surgery.

**Table 1:** Clinicodemographic characteristics of the study population

Clinical characteristics (N = 318)	Mean ± SD/number (percentage)	Min	Max
Age (years)	80.23 ± 9.29	61	99
*Sex*			
Male	97 (30.5%)		
Female	221 (69.5%)		
BMI	22.37 ± 3.85	14.0	40.8
*Fracture type*			
Femoral neck	186 (58.5%)		
Pertrochanteric	132 (41.5%)		
*Surgical methods*			
Internal fixation	182 (57.2%)		
Hemiarthroplasty	136 (42.8%)		
*Previous contralateral hip fracture*			
Yes	13 (4.1%)		
No	305 (95.9%)		
*Pre-fracture residence*			
Dwelling in a home	293 (92.1%)		
Inhabiting a care institution	25 (7.9%)		
SPMSQ score	2.98 ± 3.56	0	10
Handgrip strength (kg)	12.88 ± 8.52	0	49.3
Charlson comorbidity index score	4.75 ± 1.61	1	11
*Laboratory parameters*			
Preoperative Hb level (g/dL)	12.23 ± 1.17	7.0	16.50
Creatinine level (mg/dL)	1.22 ± 1.45	0.22	13.99
Sodium level (mmol/L; n = 316)	136.86 ± 3.88	118.0	148.0
Albumin level (g/dL; n = 292)	3.11 ± 0.36	1.8	4.4
*Surgical data*			
Surgical delay (day)	3.64 ± 9.03	0.13	124.8
Surgery duration (min)	80 ± 33	22	240
Intraoperative blood loss (cc; *n* = 314)	110 ± 105	10	750
BI score at pre-fracture	87.99 ± 19.30	0	100
BI score at 3 months	73.38 ± 27.28	0	100
BI score at 1 year	74.62 ± 30.24	0	100
EQ-5D-3L score at pre-fracture	0.88 ± 0.17	0.18	1
EQ-5D-3L score at 3 months	0.78 ± 0.22	0	1
EQ-5D-3L score at 1 year	0.79 ± 0.23	0.1	1

BMI—body mass index; SPMSQ—Short Portable Mental Status Questionnaire; Hb—hemoglobin; and SD—standard deviation; BI—Barthel Index; EQ-5D-3L, EuroQol-5D-3L

The results of the univariate analyses indicated age, BMI, fracture type (femoral neck or periprosthetic), pre-fracture residence, SPMSQ score, handgrip strength, CCI score, preoperative hemoglobin level, serum albumin level, surgical delay, and change in ADL by 3 months after surgery to be independently associated with the patients’ BI scores at the 1-year follow-up. In addition, age, fracture type, SPMSQ score, handgrip strength, CCI score, preoperative hemoglobin level, serum albumin level, surgical delay, and change in QoL score by 3 months after surgery were independently associated with the patients’ EQ-5D-3L scores at the 1-year follow-up [see Additional file 1: Table S1 ].

The multivariate linear regression analyses revealed that the changes in ADL and QoL by 3 months post-surgery from pre-fracture status were significantly associated with the corresponding outcomes at 1-year follow-up (unstandardized coefficient [*β*] = 0.019 and 0.319, respectively; *p* < 0.001 for both). The explanatory power of the BI model was reasonable (adjusted *r*^2^ = 0.417), and the explanatory power of the EQ-5D-3L was modest (adjusted *r*^2^ = 0.263). SPMSQ score was significantly and negatively associated with both BI and EQ-5D-3L score at 1-year follow-up (*β* = − 0.13 and−0.028, respectively; *p* < 0.001 for both). Furthermore, CCI score was negatively associated with ADL at 1-year follow-up (β =− 2.602; *p* < 0.001) (Table 2).

**Table 2:** Multivariate linear regression results for predicting ADL performance and QoL 1 year after hip surgery

Variable	Bata coefficient	*p* value	95% CI	
			Lower limit	Upper limit
*BI at 1-year follow-up (R*^*2*^* = 0.439; adjusted R*^*2*^* = 0.417)*				
Age (years)	0.015	0.165	-0.006	0.036
BMI	0.014	0.515	-0.029	0.058
Fracture type (Ref: FNF)	− 0.043	0.814	− 0.401	0.315
Pre-fracture residence (Ref: household)	− 0.279	0.073	− 0.584	0.026
SPMSQ score	− 0.130	** < 0.001**	− 0.183	− 0.076
Handgrip strength (kg)	− 0.003	0.828	− 0.026	0.021
Charlson comorbidity index score	− 2.602	** < 0.001**	− 4.579	− 0.625
Preoperative Hb level (g/dL)	− 0.014	0.788	− 0.120	0.091
Albumin level (g/dL)	0.439	0.086	− 0.062	0.939
Surgical delay (day)	− 0.007	0.728	− 0.001	0.001
Short-term change in BI	0.019	** < 0.001**	0.012	0.027
*EQ-5D-3L at 1-year follow-up (R*^*2*^* = 0.289; adjusted R*^*2*^* = 0.263)*				
Age (years)	− 0.004	0.191	− 0.009	0.002
Fracture type (Ref: FNF)	− 0.069	0.127	− 0.157	0.020
SPMSQ score	− 0.028	** < 0.001**	− 0.041	− 0.014
Handgrip strength (kg)	0.005	0.099	− 0.001	0.011
Charlson comorbidity index score	− 0.021	0.173	− 0.052	0.009
Preoperative Hb level (g/dL)	− 0.012	0.365	− 0.038	0.014
Albumin level (g/dL)	0.037	0.569	− 0.090	0.163
Surgical delay (day)	− 0.061	0.243	− 0.004	0.001
Short-term change in EQ-5D-3L score	0.319	**0.001**	0.125	0.513

Bold values indicate significant* P* < 0.05

Adj.B—adjustment beta coefficient: ADL—activity of daily living; BMI, body mass index; FNF—femoral neck fracture; Ref—reference; SPMSQ—Short Portable Mental Status Questionnaire; Hb—hemoglobin; BI—Barthel Index; EQ-5D-3L, EuroQol-5D-3L; QoL—quality of life, and CI—confidence interval

**Fig. 4:**
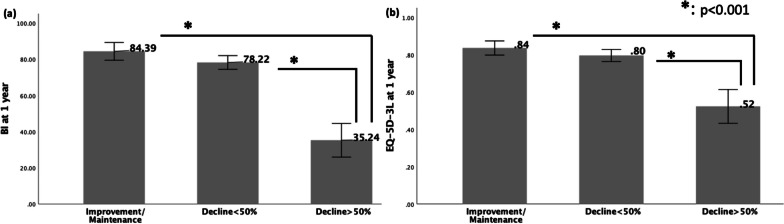
Comparison of 1-year outcomes in ADL **a** and QOL **b** among three groups based on changes in ADL and QoL three months post-surgery compared to the pre-fracture status

Figure 4 depicts the disparities in one-year outcomes following hip fracture surgery among three groups of changes in ADL and QoL three months post-surgery compared to the pre-fracture status. In the ADL category, there were 107 (33.6%) patients in the improvement/maintenance group, 169 (53.1%) in the decline < 50% group, and 42 (13.3%) in the decline > 50% group. In the QOL group, there were 136 (42.8%) patients in the improvement/maintenance group, 154 (48.4%) in the decline < 50% group, and (8.8%) 28in the decline > 50% group. The ANOVA with Bonferroni post hoc analysis revealed a significant difference in 1-year outcomes for both ADL and QOL between the improvement/maintenance group and the decline > 50% group (*p* < 0.001), as well as between the decline < 50% group and the decline > 50% group (*p* < 0.001).

## Discussion

In this prospective study, we discovered that the extent of the decline in BI and EQ-5D-3L score three months after surgery was significantly associated with the corresponding outcomes 1 year after hip fracture surgery in the older adults. Additionally, the severity of cognitive impairment, as evaluated using the patients' SPMSQ scores at admission, predicted ADL and QoL outcomes at one-year follow-up. Patients with higher comorbidities had poorer ADL outcomes one year after hip fracture surgery. Our findings suggest that the ability of a patient to withstand short-term functional decline following hip fracture surgery could influence their long-term outcomes. This underlines the significance of advanced rehabilitation services and efficient interventions for improving patients' early recovery, from a healthcare delivery and policy standpoint.

The recovery rate of older adults from hip fracture may depend on their physical resilience. Physical resilience refers to the capacity to recover physical health or to withstand functional decline resulting from physical stressors [32]. After hip fracture surgery, physical resilience has been linked to overall functional status and quality of life. Research has shown that patients with greater physical resilience have reduced disability and improved functional outcomes during rehabilitation [9, 33]. In the present studies, ADL, QoL, handgrip strength, and SPMSQ score were reported as trajectories of functional recovery and related to physical resilience[10, 34, 35]. Furthermore, another study recognized both ADL and QoL as appropriate tools for evaluating physical resilience [11]. In our study, we identified patients with minimal decline in ADL and QoL three months after surgery as being highly physically resilient, leading to better outcomes one year after the hip fracture.

Moreover, our research illuminates a swift recovery trend observed in patients with strong physical resilience following hip fracture surgery. This revelation offers valuable guidance for clinical strategies aimed at mitigating short-term functional decline and enhancing long-term outcomes for older adults. Our study has also unveiled the potential of short-term functional recovery as a predictor for long-term ADL and QoL outcomes in older adults undergoing hip fracture surgery. Additionally, in comparison with the group that showed no decline, our study identifies a noteworthy difference in 1-year outcomes for both ADL and QOL among patients experiencing a decline in ADL and QOL three months post-surgery compared to their pre-fracture status. Moreover, those with a decline exceeding 50% demonstrated even more unfavorable one-year outcomes. This underscores the significance of these clinical indicators as invaluable cues for clinicians in practical clinical assessments to forecast functional outcomes. This novel insight represents a previously unrecorded aspect in the existing literature, accentuating the critical role that early recovery plays in forecasting outcomes over the long term. It is crucial to acknowledge that these outcomes do indeed highlight variations in individual adherence to rehabilitation protocols. This variation can be attributed to the fact that our study adopted a personalized approach to rehabilitation, tailoring it to each patient's condition and expert recommendations. This diversity could potentially account for some of the observed early functional changes. To enhance short-term functional recovery following hip fracture, it appears that early postoperative rehabilitation programs could offer advantages for older adults following surgery. This notion is supported by the work of Pioli et al., who compared care plans from three medical centers for patients aged over 75 following hip fracture. They found that those who received early rehabilitation exhibited minimal functional decline six months after the fracture [36]. Consequently, our interpretation of these research findings emphasizes the significance of advanced rehabilitation services and efficacious interventions in promoting early recovery among patients. However, the specific strategies for intervention still necessitate further rigorous experimental validation.

In the present study, in addition to the short-term changes in ADL performance and QoL, cognitive impairment was identified as a predictor of these outcomes at 1 year after hip fracture surgery. After hip fracture, patients with intact cognitive function have exhibited considerably greater improvements in ADL performance compared with patients with moderate cognitive impairment [37]. Several large clinical studies have shown that cognitive impairment is a strong predictor of limited functional recovery during rehabilitation for older patients after hip fracture [38]. These findings are consistent with our finding that the SPMSQ score was negatively associated with the BI and EQ-5D-3L score at 1-year follow-up.

We found that comorbidities played a role in determining postoperative functional outcomes. The results from our multivariate analyses indicated that a high CCI score is a predictor of poor functional outcomes at 1 year after hip fracture surgery. This finding corroborates that of another study reporting that comorbidities contribute to delayed and poor functional recovery after hip fracture [39]. Furthermore, Hsiao et al. reported that patients with hip fracture who have CCI scores of ≥ 3 have a 19% higher risk of repeat osteoporotic fracture than those with CCI scores of 0 at 1-year follow-up [40]. Compared to the beta coefficients attributed to SPMSQ score and CCI, the beta coefficient corresponding to ADL and QOL exhibited relatively low values. The predictive effect for long-term outcome was modest. This implies that although the alterations in ADL and QoL three months post-surgery compared to the pre-fracture status are innovative and substantial predictive factors, baseline cognitive impairment and comorbidities exert a stronger influence than changes in ADL and QoL. Ultimately, the pre-fracture baseline status holds greater significance than postoperative dynamic changes.

Prior studies have also identified serum hemoglobin levels, serum albumin levels, handgrip strength, long surgical delays, and institutional care as predictors of functional outcomes after hip fracture surgery [15, 41, 42]. These variables demonstrated statistically significant association during the univariate analysis phase. However, the results of our multivariate linear regression analysis showed that these variables were not significantly associated with 1-year functional outcomes in older adults following hip fracture surgery. This phenomenon can be rationalized by the attenuation of their impact within the multivariate model, which accommodated the incorporation of various covariates [43].

## Limitations

This study has some limitations. First, due to the small sample size, our findings may not represent all older adults in Taiwan. Second, the functional outcomes were reported by the caregivers or patients, which may have introduced imprecision and observer biases. Third, we did not evaluate the effects of various factors of postoperative rehabilitation regimens, such as the intensity, duration, and patients’ compliance; these may be predictors of functional recovery after hip surgery and could potentially account for some of the observed early functional changes. Furthermore, we did not consider postoperative confounding factors, such as psychiatric medication use, mental state, quality of care from caregivers, and living conditions during follow-up, which are believed to affect the outcomes of hip surgery in older adults. Future studies should therefore gather more accurate data on these specific rehabilitation aspects and potential confounding factors. Finally, we studied only the 1-year postoperative functional outcomes in older adults with hip fracture. Thus, studies employing long-term follow-ups are warranted to determine the natural course of recovery and its clinical effects on functional outcomes in a similar cohort. It would be beneficial for future studies to investigate the factors contributing to functional decline up to 3 months.

## Conclusions

We demonstrated that short-term functional recovery may predict long-term ADL and QoL outcomes following hip fracture surgery in older adults. Patients who experience a smaller decline in functional ability and quality of life will have better ADL and QoL outcomes after one year.

### Supplementary Information

**Additional file 1:** Univariate regression results for factors potentially predictive of postoperative BI and EQ-5D-3L in older adults with hip fracture.

## Data Availability

The datasets analyzed during the current study are available from the corresponding author on reasonable request.

## Abbreviations

ADLs: Activities of daily living
BI: Barthel Index
BMI: Body mass index
CCI: Charlson comorbidity index
EQ-5D-3L: EuroQol-5D-3L
QoL: Quality of life
SPMSQ: Short Portable Mental Status Questionnaire
