# Supplementary material for: Minimal short-term decline in functional performance and quality of life predicts better long-term outcomes for both in older Taiwanese adults after hip fracture surgery: a prospective study
Source: J Orthop Surg Res. 2023 Oct 24;18:791. doi: 10.1186/s13018-023-04278-3 (PMC10594772; doi:10.1186/s13018-023-04278-3)
Supplement: Supplementary file 1 — Additional file 1: Univariate regression results for factors potentially predictive of postoperative BI and EQ-5D-3L in older adults with hip fracture. [file 13018_2023_4278_MOESM1_ESM.docx]

**Supplementary Table 1**. Univariate regression results for factors potentially predictive of postoperative BI and EQ-5D-3L in older adults with hip fracture.

| Variable | B | *p* value | B | *p* value |
| --- | --- | --- | --- | --- |
|  | **BI at 1-year follow-up** | | **EQ-5D-3L at 1-year follow-up** | |
| **Age** | −0.019 | <0.001 | −0.01 | <0.001 |
| **Sex (female vs. male)** | −0.008 | 0.972 | 0.021 | 0.671 |
| **BMI** | 0.051 | 0.021 | 0.008 | 0.146 |
| **Fracture** **type** (PCF vs. FNF) | −0.188 | 0.01 | −0.092 | 0.041 |
| **Surgical methods** (Internal fixation vs. Hemiarthroplasty) | 0.022 | 0.702 | 0.02 | 0.721 |
| **Previous hip fracture** (Yes vs. No) | 0.013 | 0.816 | 0.02 | 0.721 |
| **Pre-fracture residence** (home vs. care institution) | −0.46 | 0.011 | −0.076 | 0.065 |
| **SPMSQ score** | −0.188 | <0.001 | −0.043 | <0.001 |
| **Handgrip strength (kg)** | 0.046 | <0.001 | 0.014 | <0.001 |
| **Charlson comorbidity index score** | −0.193 | 0.002 | −0.069 | <0.001 |
| **Laboratory parameters** |  |  |  |  |
| Preoperative Hb level (g/dL) | 0.108 | 0.01 | 0.023 | 0.016 |
| Creatinine level (mg/dL) | 0.006 | 0.932 | −0.017 | 0.276 |
| Sodium level (mmol/L; n = 316) | 0.023 | 0.366 | 0.005 | 0.345 |
| Albumin level (g/Dl; n = 292) | 0.819 | 0.001 | 0.186 | 0.004 |
| **Surgical data** |  |  |  |  |
| Surgical delay (day) | −0.148 | 0.008 | −0.158 | 0.005 |
| Surgery duration (min) | 0.001 | 0.827 | −0.024 | 0.665 |
| Intraoperative blood loss (cc) | 0.001 | 0.54 | −0.062 | 0.274 |
| Short-term change | 0.022 | <0.001 | 0.416 | <0.001 |
| Abbreviations: BMI, body mass index; PCF, pertrochanteric fracture; FNF, femoral neck fracture; SPMSQ, Short Portable Mental Status Questionnaire; Hb, hemoglobin; BI, Barthel Index; and EQ-5D-3L, EuroQol-5D. | | | | |
